# A novel approach to control air leaks in complex lung surgery: a retrospective review

**DOI:** 10.1186/1749-8090-7-49

**Published:** 2012-06-01

**Authors:** Ara Klijian

**Affiliations:** 1 Scripps and Sharp Healthcare, 3131 Berger Ave Ste 250, San Diego, CA, 92123, USA

**Keywords:** Pleural sealant, Air leak, Lung resection, Decortications, Chest tube, Length of hospital stay

## Abstract

**Background:**

Intra-operative air leaks (IOAL) are common complications of pulmonary surgery. The post-operative management of air leaks requires a chest tube which may lead to longer hospitalization, further medical complications, and increased costs. Sealants have been shown to help control intra-operative air leaks and studies have demonstrated a reduction in chest tube duration and/or length of hospital stay. Nevertheless, systematic reviews have not presented sufficient evidence to recommend their general use in lung resection.

**Methods:**

One hundred and twenty-one consecutive patients who underwent pulmonary surgery with and without Progel® Pleural Air Leak Sealant were reviewed retrospectively. Intra-operative and 3-months postoperative data were assessed for the presence and persistence of air leaks, chest tube duration, the length of hospital stay, and complications.

**Results:**

Seventy patients (57.9%) had IOAL. Thirty-six were treated with Progel in addition to standard intra-operative technique (pleural-sealant group; PSG) and 34 patients were treated only with standard technique (control group; CG). The percentage of post-operative air leaks in the PSG was 11% (1.2% >Grade 2 air leak) compared with 58.8% (6% >Grade 2 air leak) in the CG (*p* <0.0001, Leaks graded from 1 = small air leak to 7 = large air leak). The median chest tube duration was significantly shorter in the PSG compared with the controls (1.0 versus 2.5 days; *p* < 0.0001). The median length of hospital stay was 50% lower in the PSG compared with the control group (1.5 versus 3.0 days; *p* = 0.047). There were no significant differences in complications between the two groups.

**Conclusions:**

The results of this single-center, single surgeon, retrospective review demonstrate a significant reduction in IOAL, chest tube duration, and length of hospital stay in the in patients treated with Progel when compared with standard intra-operative closure management alone. They suggest that the use of a pleural sealant is more effective in reducing alveolar air leaks associated with lung resection compared with standard closure techniques alone and may result in both an improved surgical outcome and a reduction in costs associated with prolonged hospital stay.

## Background

Intra-operative alveolar air leaks are common complications associated with pulmonary resection and other intra-thoracic procedures requiring extensive pleural dissection [1]. The intra-operative air leak (IOAL) rate has been reported to be between 48% and 75%, with persistence beyond 7 days in 15%-18% in patients experiencing complications resulting in prolonged air leaks [2-7]. Managing these air leaks requires insertion of a chest tube and longer hospitalization, which potentially could increase morbidity and post-operative costs. Therefore, controlling IOAL can add benefits to the patient, reduce chest tube duration, length of hospital-stay and associated morbidities, and potentially reduce healthcare cost.

Lung sealants have been shown to help control IOAL’s with a few studies demonstrating a reduction in chest tube duration and/or length of hospital stay [1,2,8-10]. Various lung sealant materials have been developed in the past two decades including fibrin-based materials [9,10], synthetic [5] and fleece-bound sealants [3]. We report here our experience with the FDA-approved pleural air leak sealant Progel® (Neomend, Inc. Irvine, CA), for the treatment of air leaks incurred during open thoracotomy after standard visceral pleural closure. Progel is a polymeric biodegradable hydrogel sealant which is clear, flexible, and adheres well to the lung tissue [11]. We initiated the use of Progel during lung resections in our institution in May 2010. Based upon the clinical experience at our institution and preclinical study, Progel is biodegradable and is completely reabsorbed from the lung surface within 14 days following surgery [11].

## Methods

Between May 2009 and August 2010, we performed a retrospective chart review of prospectively collected data on 121 consecutive patients who underwent lung surgery with and without Progel Pleural Air Leak Sealant application. Preoperative, operative and 3-months postoperative data were reviewed for all included patients. Patient inclusion criteria included patients 18 years or older who underwent lung resection including lobectomy, segmentectomy, wedge resection, and decortications with and without Progel application. There were no exclusion criteria. Patients treated without Progel were selected before May 2010, the time when Progel became available in our institution. The study protocol was reviewed and approved by a Central Institutional Review Board (Chesapeake IRB, Columbia, MD).

### Study endpoints

Patient charts were assessed for the following endpoints: presence of intra-operative air leaks, postoperative air leaks, median chest tube duration, and the mean and median length of hospital stay. In addition, intra-operative and postoperative complications were reviewed and analyzed. All patients were followed in the clinic for a minimum of 3 months after surgery.

### Statistical analyses

For continuous variables, statistical analyses included calculation of mean, median, range and standard deviation values. Binary variables are presented as simple proportions. Proportions for baseline study characteristics, surgical procedures, and postoperative air leaks were compared between groups using the chi-squared or Fisher’s exact tests to check for equality. A nonparametric test was used to compare medians. Mean chest tube duration and hospital length of stay (LOS) between groups were compared using the two-sample t-test. P-values were used to determine significance at the 5% level. All analyses were performed using SAS®, version 9.1.

### Progel application

In those patients treated with Progel (supplied by Neomend, Inc. Irvine, CA), upon observing an IOAL, corrective surgical measures were undertaken initially, followed by Progel application. For sutures or staple lines, the general technique for Progel application was to initially apply a discrete focal line and, after approximately one minute, to reapply Progel in a mist configuration to incorporate the surrounding 2–3 inch area of tissue. In decortication patients, the technique was predominately to apply mist coverage to the entire decorticated surface. In all cases a single one vial (4 mL) Progel kit was used.

## Results

One hundred and twenty one surgical patients with lung procedures in our clinic met the criteria for study inclusion. Sixty patients were treated with Progel Pleural Air Leak Sealant (PSG) and sixty-one patients without the sealant (CG). The mean and median patient age at the time of surgery was 64.5 ± 12.0 and 67 years, respectively, (range, 19 to 89). The study population included 45.5% Caucasians, 32.2% Hispanics, 13.2% African-American, and 9.1% other. Demographics and baseline data for both groups are shown in Table 1. Patient characteristics with regard to age, gender, and comorbidities showed no significant differences between both groups with the exception of patients with preoperative bleb/pneumothorax. These were significantly higher in the pleural sealant group. Surgical procedures performed in 121 patients included 54 single wedge resections, 33 decortications, 19 lobectomies, 11 segmentectomies/bisegmentectomies and 4 other procedures. In addition to malignancies, pathologies included pulmonary nodules of unknown etiology, open surgical biopsies, diagnosis of interstitial disease and infection of bacterial and fungal origin were indicated for the procedure. In the decortication procedures, the majority were due to empyema, including, in both the PSG and CG, those with active infection and those with malignant effusion and tumor peel. The distribution of the surgical procedures in both groups (Table 2) was similar with the exception of segmentectomy/bisegmentectomy procedures which were higher in the pleural-sealant group. 

**Table 1:** Baseline study characteristics

	**Pleural Sealant Group**	**Control Group**	***p*value**
	**N = 60**	**N = 61**	
**Males**	34 (56.7%)	37 (60.7%)	0.6559
**Females**	26 (43.3%)	24 (39.3%)	0.6559
**Median age in years (range)**	68 (25 – 89)	64 (19 – 83)	0.1224
**COPD**	20 (33.3%)	20 (32.8%)	0.9491
**Emphysema**	8 (13.3%)	10 (16.4%)	0.6362
**TB / Hx of TB**	3 (5%)	3 (4.9%)	0.6523
**Bleb / Pneumothorax**	5 (8.3%)	0	**0.0275**
**Smoking**	48 (80%)	40 (65.6%)	0.0748
**Prior RT**	11 (18.3%)	12 (19.7%)	0.8511
**Malignancy resection**	19 (31.7%)	13 (21.3%)	0.1966

**Table 2:** Surgical procedure

	**Pleural Sealant Group**	**Control Group**	***p*value**
	**N = 60**	**N = 61**	
**Total single wedge resection**	24 (40%)	30 (49.2%)	0.3098
**Total decortication**	17 (28.3%)	16 (26.2%)	0.7950
**Lobectomy**	8 (13.3%)	11 (18%)	0.4775
**Segmentectomy/ Bisegmentectomy**	9 (15%)	2 (3.3%)	**0.0251**
**Other (Blebs, Lingula, Grillo)**	2 (3.3%)	2 (3.3%)	0.6844

After lung resection, 70 (57.9%) patients had IOALs, of which 6% were a Grade 2 or larger. Patients in the pleural sealant group (n = 36) were treated with Progel while in the control group (n = 34), no further intervention was performed. No sealant application of any type was used in in the control group. There were no differences in the postoperative management of patients in either group.

Of the remaining patients without IOALs, there were 24 patients in the pleural-sealant group where Progel was applied on fragile and weak tissue area due to concern of potential air leaks, and 27 patients in the control group with no further intervention. Chest tubes were inserted in all patients in both groups to manage air leaks and for drainage. The location and distribution of IOAL in both groups (Table 3) were not significantly different.

**Table 3:** Intraoperative air leak locations

	**Pleural Sealant Group**	**Control Group**	***p*value**
	**N = 36**	**N = 34**	
**Parenchyma**	19 (52.8%)	16 (47%)	0.6324
**Staples line**	11 (30.6%)	14 (41.2%)	0.3540
**Adhesions/ re-do**	6 (16.6%)	4 (11.8%)	0.4051

### Postoperative air leak

**Figure 1:**
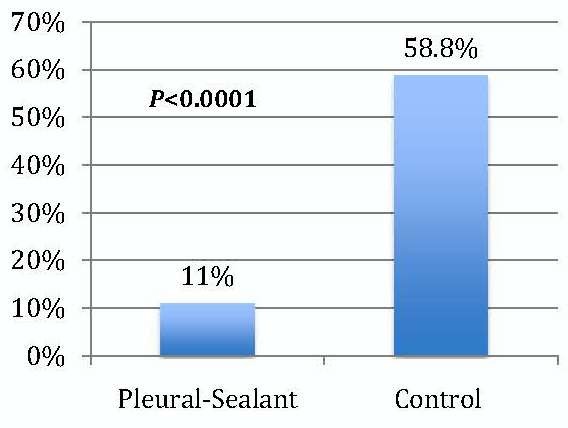
Patients with Postoperative Air Leak.

Postoperative air leaks in patients in the PSG with IOAL was significantly reduced (Figure 1). Only 11% (4/36) experienced postoperative air leaks, of which 1.2% were a Grade 2 or larger leak, compared to 58.8% (20/34) in the control group (p <0.0001).

With regard to those patients with no IOAL, none of the twenty-four patients in the PSG had a postoperative air leak, whereas 7.4% (2/27) of the CG patients had a postoperative air leak. This difference did not reach statistical significance (*p* = 0.08).

### Chest tube duration

**Figure 2:**
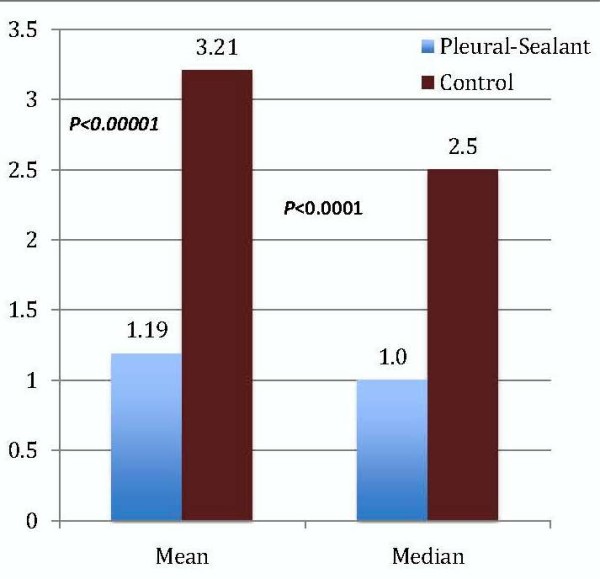
Mean and Median Chest Tube Duration (days).

The duration of chest tube drainage was significantly reduced in the pleural sealant group. The difference between the pleural sealant group and control group in both mean and median chest tube duration values was significantly different (Figure 2). The mean chest tube duration was 1.19 ± 0.52 days in the pleural sealant group compared with 3.21 ± 2.14 days in the control group, (*p* < 0.00001). The median chest tube duration was 1.0 day (range, 1 to 3) in the pleural sealant group compared with 2.5 days (range, 1 to 7) in the control group (p < 0.0001).

In patients with no IOAL, the chest tube duration was similar in both groups. The mean and median chest tube durations were 1 day in both groups.

### Length of hospital stay

**Figure 3:**
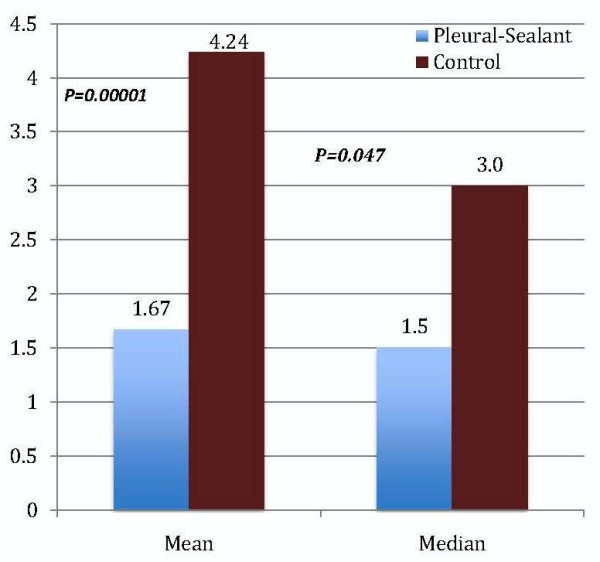
Mean and Median Length of Hospital Stay (days).

The length of hospital stay (LOS) was significantly reduced in the pleural sealant group (Figure 3). The mean LOS was 1.67 ± 0.83 days in the PSG and 4.24 ± 2.13 days in the CG (*p* = 0.00001). The median values were 1.5 days (range, 1 to 4) and 3.0 days (range, 2 to 9) in the PSG and CG respectively, (*p =* 0.047).

In patients with no IOAL, the LOS was lower in the pleural sealant group but did not reach statistical significance. The mean and median was 1.38 ± 0.71 and 1.0 (range, 1 to 3) days in the PSG, and 2.48 ± 1.22 and 2.0 (range, 2 to 8) days in the CG, respectively. When all patients, with and without IOAL, were analyzed, the LOS was significantly favoring the PSG. The median LOS in the PSG was 1.0 (range, 1 to 4) days versus 3.0 (range, 2 to 9) days (p < 0.00001).

### Complications

There was no statistically significant difference in complications between the treatment and control groups. Five (8.3%) post-operative complications occurred in the pleural sealant group compared to eight (13.1%) in the control group. There were three patients (5%) with atrial fibrillation, one patient (1.7%) with urinary tract infection (UTI) and one patient (1.7%) with phlebitis in the pleural sealant group versus three patients (4.9%) with atrial fibrillation, two patients (3.3%) with UTI, two patients (3.3%) with pneumonia, and one patient (1.6%) with pneumothorax in the control group. There were no other complications reported.

## Discussion

Intra-operative air leak rates after lung resection have been reported to be between 48% to 74% [1-4], with persistence beyond 7 days in 15-18% [6,7]. Controlling IOAL and subsequent postoperative air leaks is desirable and often leads to a reduction in chest tube duration, length of hospital stay, and associated morbidities. The use of hydrogel sealants as an adjunct to the standard surgical procedures, suturing, stapling, buttressing, and electrocautery, to seal IOALs after lung surgery has been investigated for the past 2 decades. Although many of these sealants have been shown to control IOALs, few studies have demonstrated reduction in post-operative air leaks, chest tube duration, and/or in length of hospital stay [1-5,8-10].

In studies of the use of lung sealants to control IOAL we speculated that inter-surgeon variations in air leak closure techniques and experience among different surgeons and different surgical centers may contribute to some degree of the statistical variability reported in comparing the efficacy of sealants with standard closure methods. This may lead to inconsistent or inconclusive results among ostensibly similar clinical studies and to the conclusion that lung sealants are not sufficiently effective to be recommended as a standard of care in lung resections [1,12]. In an effort to reduce the degree of surgical variability in technique as a confounding study variable, we conducted a single-site, single-surgeon, retrospective study of pulmonary resection results in which we compared clinical outcomes in patients treated with an intra-operative surgical sealant and those in whom IOAL were closed using standard closure methods. Our results have demonstrated a significant reduction in IOAL, postoperative air leak, chest tube duration, and hospital length of stay in the patients treated with Progel, a hydrogel sealant, to close intra-operative air leaks in comparison with the patients in whom intra-operative leaks were closed with standard closure techniques.

Additionally, when compared with data from a prospective, randomized, multicenter, clinical trial of Progel in addition to standard closure techniques in patients undergoing lung resection in comparison with standard techniques alone, we show a more statistically significant difference between treated and control patients with respect to chest tube duration and length of hospital stay [2]. In the prospective study of a 161 patients with IOAL, Allen et al demonstrated significant air leak reduction in the sealant group. Thirty-five percent (35%; 36/103) of patients in the sealant group compared to 14% in the control group remained air leak free through one month follow up (*p* = 0.005). They also demonstrated significant reduction in LOS with a median of 6 days in the sealant group versus 7 days in the control group (*p* = 0.028). Chest tube duration was similar in both groups at a median of 6.8 days in the sealant group versus 6.2 days in the control group [2] In our study we showed a significant difference in mean chest tube duration between treatment and control patients, 1.19 days versus 3.21 days, respectively (*p* < 0.00001). We showed a mean LOS difference between treatment and control groups of 2.6 days (*p* = 0.00001). There was a notable difference between the two study populations with respect to the predominant type of lung resection (54% single wedge resection in our study versus 58% lobectomy in the Allen et al trial). However, this difference is not considered to prevent a comparison between the two trials because, in a retrospective review of risk factors associated with postoperative pulmonary complications in 266 lung resection patients, where prolonged air leak, defined as >7 days, was one of the predominant complications, the type of lung resection was not found to be a significant risk factor [13].

In other studies, D’Andrilli et al. [14] randomized 203 patients to evaluate the efficacy of a synthetic lung sealant compared with standard closure techniques. They showed a significant reduction in IOALs (85.3% in sealant group versus 59.4% in the control, *p* < 0.001), a significant reduction in air leak duration but no report on chest tube duration and no significant difference in median LOS (5 days median in the sealant group versus 6 days in control) [11]. In a recent similar study of 299 randomized patients, Marta et al demonstrated a significant reduction in IOAL (71% in the sealant group versus 62% in the control, *p* = 0.042), a reduction in median chest tube duration (4 days in the sealant group versus 5 days in the control group; p = 0.054), and no difference in median LOS (8 days in the sealant group versus 9 days in the control group; *p* = 0.35) [4] In a single center study, Tansley et al randomized 52 patients with IOAL to evaluate a bovine serum albumin/glutaraldehyde sealant [15]. They reported a significant reduction in median duration of post-operative air leaks (1 day in the sealant group versus 4 days in the control; *p* < 0.001), median chest tube duration (4 days in the sealant group versus 5 days in the control group; *p* = 0.012), and median LOS of 6 days in the sealant group versus 7 days in the control (*p* = 0.004) [15].

The results in this investigation, including a low post-operative air leak rate of 11%, a one-day median chest tub duration, and a median 1.5 days LOS in the pleural sealant group, demonstrate significant reduction in key clinical outcomes when compared with the control group and are favorable when compared with published studies of Progel and other lung sealants. In addition, there was no difference in complication rates during the surgery and postoperatively up to 3 months follow up.

While this study did not address directly the issue of cost of care, an implication of the reduction in the length of stay observed in the pleural sealant group is that healthcare costs would be reduced in association with sealant use. Handy et al have recently reported that reductions in the length of stay following lobectomy or pneumonectomy result in substantial cost-of-care savings [16].

During surgery, the pleural-sealant is easy to prepare and use. It conforms and adheres to the tissue surface area well without causing any tissue trapping or restriction when the lung is inflated.

A limitation of this study is its non-randomized, retrospective design. However, by design, strengths include the use of single surgeon and prospectively collected data. In addition, the lack of extensive inclusion and exclusion criteria suggest that the results represent a real practice picture for the treatment of IOALs after lung surgery. The results of this study appear to suggest that other studies of lung resection patients in single-center and, possibly single-surgeon, consecutive-patient or case–control groups in which lung sealant is used in comparison with standard air leak closure techniques are warranted to elucidate the possible role of variations in surgical techniques or the methods of lung sealant use in explaining the degree of clinical efficacy of lung sealants.

## Conclusions

The results of this retrospective review demonstrate a significant reduction in IOAL, postoperative air leaks, chest tube duration and length of hospital stay in the pleural sealant group in comparison with the standard management of air leaks during lung surgery. This investigation suggests that Progel Pleural Air Leak Sealant is effective and safe, and supports and extends prior published clinical data. The reduction in IOAL can add benefits to the patient, reduce chest tube duration, length of hospital-stay and associated morbidities, and potentially reduce healthcare cost. Further prospective randomized studies with focus on chest tube duration and hospital length of stay may add further value and confirm our results.

## Abbreviations

CG: Control group; IOAL: Intra-operative air leak(s); PSC: Pleural sealant group.

## Competing interests

The author reports no competing interests.

## Author’s contributions

The author designed the study protocol, performed all surgeries and all chart reviews, wrote the manuscript, and reviewed and approved of the edited manuscript.

## Author information

Dr. Ara S Klijian MD practices cardiothoracic surgery, vascular surgery and critical care surgery in San Diego, California.
